# Lactoferrin concentration in breast milk during the first postpartum year in Lima Norte, Peru

**DOI:** 10.17843/rpmesp.2026.433.16525

**Published:** 2026-09-25

**Authors:** Edison A. Rivera-Fernandez, David Durand, Rafaella Navarro, Yamile Rodriguez-Angeles, Pia S. Pannaraj, Theresa J. Ochoa

**Affiliations:** 1 Instituto de Medicina Tropical “Alexander von Humboldt”, Universidad Peruana Cayetano Heredia, Lima, Peru. Instituto de Medicina Tropical “Alexander von Humboldt” Universidad Peruana Cayetano Heredia Lima Peru; 2 Laboratorios de Investigación y Desarrollo, Universidad Peruana Cayetano Heredia, Lima, Peru. Laboratorios de Investigación y Desarrollo Universidad Peruana Cayetano Heredia Lima Peru; 3 Division of Infectious Diseases, Department of Pediatrics, University of California, San Diego; La Jolla, CA, United States. University of California Division of Infectious Diseases Department of Pediatrics University of California San Diego CA USA; 4 Division of Infectious Diseases, Rady Children’s Hospital, La Jolla, CA, United States. Division of Infectious Diseases Rady Children’s Hospital La Jolla CA United States

**Keywords:** Lactoferrin, ELISA, Human Milk, Breastfeeding, Postpartum Period

## Abstract

Lactoferrin (LF) is a glycoprotein with antimicrobial and immunomodulatory properties, present in breast milk and fundamental in infant protection. The objective was to describe the LF concentration during the first postpartum year in Lima Norte, Peru. A retrospective observational study was conducted based on a secondary analysis of 158 milk samples from 83 mothers: 39 from transitional milk (7-14 days) and 119 from mature milk (2-12 months). LF was quantified by ELISA. The median LF in transitional milk was 6.52 (IQR: 5.16-8.15) mg/mL and in mature milk 1.01 (IQR: 0.80-1.57) mg/mL. In the mature milk periods, the values were 3.33 mg/mL (2 months), 3.27 (3-5 months), 2.56 (6-7 months), and 2.08 (8-12 months). In the 22 mothers with paired samples, the mean concentration was 7.71 ± 2.27 mg/mL in transitional milk and 3.76 ± 1.06 mg/mL at 2 months. The absence of sufficient colostrum samples and the unequal sample availability limit the complete characterization of the trajectory during the first year. The results describe a decreasing pattern of LF concentration during the first year of lactation.

## INTRODUCTION

Breast milk is recognized as the ideal food for infant growth and development, due to its content of bioactive components such as α-lactalbumin, β-casein, secretory immunoglobulin A (sIgA), lactoferrin (LF), and lysozyme ^1^. The concentration of these protein biocomponents tends to decrease throughout the different stages of lactation: colostrum, produced between the first and fifth day, presents the highest concentrations; these decrease in transitional milk (7-14 days) and reach their lowest levels in mature milk, produced after fifteen days postpartum ^2^. Among these proteins, LF is one of the most abundant and also presents variations in its concentration according to the lactation stage ^3^^-^^6^. Its high affinity for iron limits its availability for microorganisms, inhibiting their growth and conferring a bacteriostatic effect. This mechanism, based on the sequestration of Fe²⁺ and its interaction with negatively charged structures, has been widely demonstrated in different experimental models.

Several studies have evidenced its activity against bacteria such as *Escherichia coli* and *Helicobacter pylori*, as well as in cell models infected with *Legionella pneumophila* and *Mycobacterium tuberculosis*^7^^).^ Furthermore, its activity has been documented both *in vitro* and *in vivo*, showing effects against gram-positive and gram-negative bacteria, and even against potentially antibiotic-resistant strains, such as *Listeria monocytogenes*, *Klebsiella pneumoniae*, *Salmonella enterica* and *Staphylococcus aureus*^8^^-^^10^. Beyond its antimicrobial action, LF plays a key role in the protection of neonates against infections and in the development of the immune system. Additionally, it contributes to the balance of the gut microbiota, consolidating its role as an essential component of breast milk ^11^. The physicochemical composition of breast milk varies during its maturation and can be influenced by maternal and socioeconomic factors, particularly the mother's diet and health status ^12^.

Although previous studies have described variations in LF concentration according to lactation stage, the available evidence corresponds to different populations and geographical contexts, so their results are not necessarily extrapolable to the Peruvian population. In Peru, evidence on LF concentration in breast milk during the first postpartum year is limited, particularly in samples of transitional milk and mature milk. Therefore, it is pertinent to descriptively characterize LF concentration during this period in mothers attended in Lima Norte.

In this context, the present study aimed to describe the concentration of LF in breast milk samples from full-term mothers (≥37 weeks of gestation) during the first postpartum year, differentiating between transitional milk and mature milk. Maternal, neonatal, and socioeconomic characteristics were described as population characterization variables.

**KEY MESSAGES**

Motivation for the study. Lactoferrin is an essential component of breast milk with important immunological functions. Although there are previous studies on its variation according to the stage of lactation, evidence in the Peruvian context is limited.

Main findings. A higher concentration of lactoferrin was observed in transitional milk samples compared to mature milk samples.

Public health implications. These findings provide descriptive information on lactoferrin concentration in transitional milk and mature milk in the population served in Lima Norte. The absence of colostrum samples limits the complete characterization of lactoferrin variation from the early stages of lactation.

## THE STUDY

An observational, descriptive, and retrospective study was performed on breast milk samples from mothers in Lima Norte, Peru. The study corresponds to a secondary analysis of breast milk samples from a previous multicenter study ^13^.

The study was conducted between March 2024 and February 2025 at the laboratory of the Pediatric Infectology Research Unit (InfectoPed) of the Alexander von Humboldt Institute of Tropical Medicine and at the Research and Development Laboratories of the Universidad Peruana Cayetano Heredia, located in the district of San Martín de Porres, Lima, Peru.

Eighty-three mothers were included, with a total of 158 samples: 39 of transitional milk (7-14 days postpartum) and 119 of mature milk (2-12 months postpartum). The availability of colostrum samples was limited due to their use in the previous multicenter study, so the analysis was restricted to transitional milk and mature milk.

Participants from the original study who authorized the use of their biological samples and data for secondary analyses were included. Those who did not provide such authorization were excluded.

The main variable was lactoferrin (LF) concentration in human milk, expressed in mg/mL and determined by the ELISA technique. As characterization variables, the maternal milk stage (transitional milk and mature milk), maternal factors (maternal age, gestational age, number of pregnancies, type of delivery, and body mass index), neonatal factors (birth weight, birth length, and sex), and socioeconomic factors (number of inhabitants per household, availability of basic services, area of residence, nationality, and access to the basic food basket) were considered.

For the analysis, maternal age was categorized into ≤19, 20-34, and ≥35 years; gestational age into 37-38 weeks (early term), 39-40 weeks (full term), and 41 weeks (late term) ^14^; number of gestations into 1, 2, and ≥3; type of delivery into vaginal and cesarean; and pregestational body mass index into <18.5, 18.5-24.9, 25-29.9, and ≥30 kg/m² ^15^. Birth weight was categorized into 2.5-<4.0 kg and ≥4.0 kg ^16^, while birth length was classified into <48, 48-50, and >50 cm. Maternal educational level was categorized into primary, secondary, higher, and unspecified; sex into female and male; nationality into Peru and Venezuela; availability of basic services into yes and no; and number of inhabitants per household into 1-2, 3-4, and ≥5 people. For the economic component, the household's monthly per capita income was calculated by dividing the monthly income by the number of household inhabitants and compared with the reference value of the basic food basket established by the Instituto Nacional de Estadística e Informática (INEI), being classified as «sí cubre» when the per capita income was ≥260 soles and as «no cubre» when it was <260 soles ^17^.

Breast milk aliquots were thawed and subjected to two differential centrifugations to obtain milk whey. The first centrifugation was performed at 1200 × g for 15 minutes at 4 °C, after which the aqueous fraction was separated. This fraction was subjected to a second centrifugation at 3000 × g for 15 minutes at 4 °C to obtain the milk whey, which was stored at −70 °C until analysis.

LF concentration was determined by enzyme-linked immunosorbent assay (ELISA, Assaypro, USA), following the manufacturer's instructions. Human milk whey samples were diluted to a final dilution of 1:800,000 and analyzed in duplicate, along with standards prepared by serial dilutions (20-0.313 ng/mL). Absorbance was measured at 450 nm using a microplate reader (Synergy H1m, BioTek Instruments, USA), and concentrations were calculated by interpolation from a standard curve fitted with a four-parameter logistic model (4PL), using MyAssays software (version R10.2), expressed in mg/mL.

Statistical analysis was performed using R (v4.3.2). Continuous variables were described using median and interquartile range (IQR), and additionally, the mean with its 95% confidence interval (95% CI) was reported for LF concentration. Categorical variables were summarized using absolute and relative frequencies. LF concentration was described according to lactation stage and postpartum period, considering available samples.

The study was approved by the Institutional Ethics Committee of the Universidad Peruana Cayetano Heredia (SIDISI: 213718). The samples used in the present secondary analysis came from a multicenter study (SIDISI: 205022), in which the participants provided informed consent for the collection, storage, and future use of their biological samples. For the present analysis, the information was handled confidentially and the data were used without personal identifiers.

## FINDINGS

83 mothers from Lima Norte were included, who provided a total of 158 breast milk samples during the first postpartum year. Of the total participants, 31 (37.3%) provided a single sample and 52 (62.6%) two or more, allowing for a longitudinal analysis. In relation to the lactation stage, 39 samples of transitional milk (7-14 days) and 119 of mature milk (2-12 months) were analyzed. Colostrum samples were not available due to limitations in the collected volume. Among the mature milk samples, 53 corresponded to 2 months ±15 days, 9 to 3-5 months ±15 days, 39 to 6-7 months ±15 days, and 18 to 8-12 months ±15 days. In total, 32 mothers provided samples in both lactation stages.

The mean maternal age was 27.7 ± 5.9 years, the predominant group being 20-34 years (75.9%). The mean gestational age was 38.8 ± 1.0 weeks, with a predominance of 39-40 weeks (61.4%), and vaginal delivery was the most frequent (92.8%). The mean pregestational body mass index (BMI) was 26.1 ± 5.0 kg/m², with a predominance of the normal category (44.6%), followed by overweight (33.7%). 51.8% of newborns were male, with a mean birth weight of 3.3 ± 0.4 kg and a mean height of 49.1 ± 1.4 cm. Regarding socioeconomic characteristics, secondary education predominated (50.6%), as did Peruvian nationality (89.2%), the availability of water and sewage (90.4%), and households with 1-2 inhabitants (21.7% among households with a specified number of inhabitants). According to the monthly per capita income relative to the reference value of the basic food basket, 49.4% of households were classified as "covers" and 50.6% as "does not cover" (table 1).

**Table 1:** Maternal, neonatal, and socioeconomic characteristics.

Characteristic		n=83	%
Maternal Age (years)			
	≤19	9	10.8
	20-34	63	75.9
	≥35	11	13.3
Gestational age (weeks)			
	37-38 (early term)	31	37.4
	39-40 (full term)	51	61.4
	41 (late term)	1	1.2
Number of gestations			
	1 gestation	14	16.9
	2 pregnancies	36	43.4
	≥3 pregnancies	33	39.7
Type of delivery			
	Vaginal	77	92.8
	Cesarean	6	7.2
Pregestational BMI (Kg/m²)			
	<18.5 (underweight)	2	2.4
	18.5-24.9 (normal)	37	44.6
	25-29.9 (overweight)	28	33.7
	≥30 (obesity)	16	19.3
Education of the mother			
	Primary	14	16.8
	Secondary	42	50.6
	Superior	15	18.1
	Not specified	12	14.5
Sex of the child			
	Female	40	48.2
	Male	43	51.8
Birth weight (kg)			
	2.5-<4 (normal)	81	97.6
	≥4.0 (macrosomia)	2	2.4
Birth length (cm)			
	< 48	13	15,7
	48-50	56	67.5
	>50	14	16.8
Nationality			
	Peru	74	89.2
	Venezuela	9	10.8
Water and sewerage			
	Yes	75	90.4
	No	8	9.6
Inhabitants per dwelling			
	1-2	18	21.7
	3-4	16	19.3
	≥5	11	13.2
	Not specified	38	45.8
Basic food basket			
	Yes, it covers	41	49.4
	Does not cover.	42	50.6

The LF concentration showed a progressive decrease throughout the lactation period (figure 1A). In transitional milk, the median was 6.52 mg/mL (IQR: 5.47-8.22), while in mature milk it was 1.01 mg/mL (IQR: 0.80-1.57). In the different mature milk periods, the medians were 3.33 mg/mL (IQR: 3.05-4.10) at 2 months ±15 days, 3.27 mg/mL (IQR: 2.93-4.58) at 3-5 months, 2.56 mg/mL (IQR: 2.09-3.23) at 6-7 months, and 2.26 mg/mL (IQR: 1.36-3.28) at 8-12 months (table 2; figure 1B).

**Figure 1:**
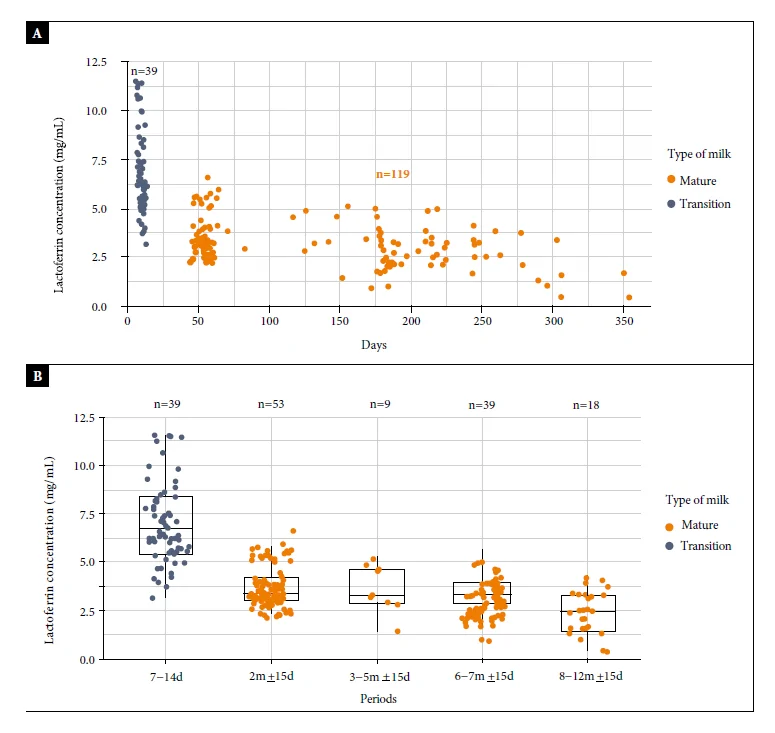
Distribution of lactoferrin concentration according to: A) postpartum days and B) lactation stages, in breast milk samples (n=158).

**Table 2:** Lactoferrin concentration (mg/ml) in transitional and mature milk at different postpartum periods (n=158).

Type of milk	n	Lactoferrin concentration (mg/mL)			
		Median (IQR)	Mean (95% CI)	Min	Max
Transitional milk (7-14 days)	39	6.52 (5.47-8.22)	7.02 (6.30-7.73)	3.15	11.49
Mature milk (2 months ±15 days)	53	3.33 (3.05-4.10)	3.72 (3.42-4.01)	2.17	5.94
Mature milk (3-5 months ±15 days)	9	3.27 (2.93-4.58)	3.64 (2.70-4.57)	1.43	5.11
Mature milk (6-7 months ±15 days)	39	2.56 (2.09-3.23)	2.73 (2.42-3.04)	0.88	4.98
Mature milk (8-12 months ±15 days)	18	2.26 (1.36-3.28)	2.24 (1.66-2.81)	0.43	4.08

IQR: interquartile range, 95% CI: 95% confidence interval.

In 32 mothers with multiple samples (n=126), a progressive decrease in LF concentration was observed from transitional milk to the different mature milk periods (Figure 2). Of these 32 mothers, 22 had a transitional milk sample and a mature milk sample corresponding to the 2-month period ±15 days (Figure 3A), with a total of 44 samples for paired analysis (Figure 3B). In this subgroup, the mean LF concentration was 7.71 ± 2.27 mg/mL in transitional milk and 3.76 ± 1.06 mg/mL in mature milk. The medians were 7.28 and 3.42 mg/mL, respectively. 59.1% of mothers showed a decrease ≤50%, while 40.9% showed a decrease >50% (supplementary material Table S1).

**Figure 2:**
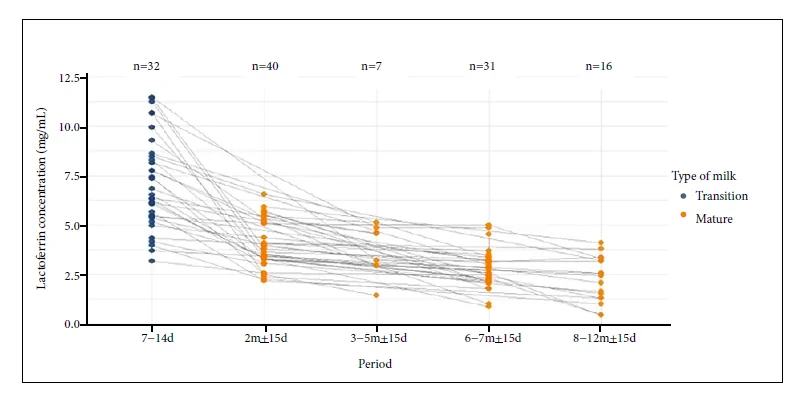
Lactoferrin concentration (mg/mL) in transitional and mature milk in mothers with longitudinal follow-up (n=126 paired samples from 32 mothers).

**Figure 3:**
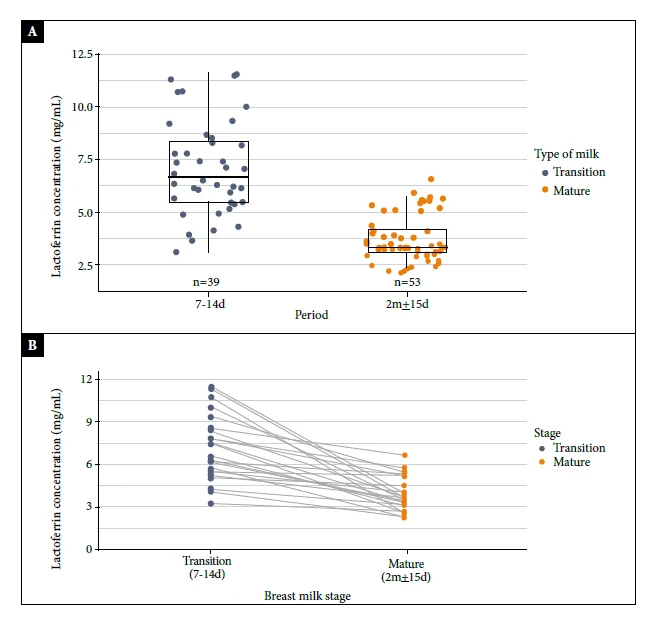
Comparison of lactoferrin concentration (mg/mL) between transitional milk and mature milk (2 months ±15 days). A) Comparison between independent groups of transitional milk (n=39) and mature milk (n=53). B) Paired comparison in 22 mothers with one transitional milk sample and one mature milk sample (22 pairs; n=44 samples).

The descriptive analysis of LF concentrations according to maternal, neonatal, and socioeconomic characteristics is presented in supplementary material table S2.

## DISCUSSION

In this study, a progressive decrease in LF concentration was evidenced during the first postpartum year, from transitional milk to mature milk. This pattern has been widely described in the literature as part of an adaptive process of breast milk, aimed at responding to the changing immunological requirements of the infant ^2^^,^^3^.

This phenomenon has also been documented in populations of premature neonates, where initial LF concentrations tend to be higher, especially in mothers of newborns and premature infants, as a compensatory mechanism against neonatal immunological immaturity ^18^^,^^19^. The magnitude of the concentrations may vary depending on the characteristics of the population.

When comparing these results with other studies, it is necessary to consider the differences between the studied populations. Turin *et al*. reported considerably higher concentrations of LF in both transitional milk and mature milk; however, their population was mainly composed of mothers of premature and low-birth-weight newborns, conditions associated with higher LF concentrations ^20^^,^^21^. In contrast, the present study included mothers of full-term newborns. This difference limits direct comparability between both populations.

In populations of full-term newborns, the findings are more consistent with our results. Mastromarino *et al*. and Hernández Pérez documented initial concentrations between 5.4 and 7.0 mg/mL, with values in mature milk between 1.7 and 2.3 mg/mL ^4^^,^^22^, ranges similar to those observed in our population in later stages. Likewise, Zhang *et al*. reported a progressive decrease during the first months of lactation, although with lower absolute values, possibly due to differences in units of measurement, analytical techniques, or population characteristics. Although the magnitude differs, the downward trend is comparable ^23^.

In our case, the reduction from 6.52 mg/mL in transitional milk to 2.26 mg/mL between 8 and 12 months evidences the dynamic nature of LF concentration during lactation ^3^. Furthermore, longitudinal studies have described that, after this initial decrease, LF may stabilize or even slightly increase in later stages of lactation ^24^.

Various studies have described variations in LF concentration in relation to maternal, neonatal, and contextual characteristics ^(^^25^^-^^27^. However, the differences in the populations studied, the time of sample collection, and the methodologies employed make it difficult to establish direct comparisons with the descriptive results of the present study.

In this context, other studies provide complementary evidence. Czosnykowska-Łukacka *et al*. observed increases in LF in prolonged lactations ^28^, while Kulesza-Brończyk *et al*. reported variations in LF concentration according to neonatal characteristics in early stages ^29^. For their part, Rai *et al*., in a systematic review, described higher LF concentrations in initial stages and a subsequent decrease to values corresponding to mature milk ^30^.

Among the study's limitations are the small sample size and the population's relative homogeneity. Additionally, not all mothers provided samples at all stages, leading to an unequal distribution of observations across postpartum periods and limiting their representativeness. Furthermore, potentially relevant variables, such as maternal diet and inflammatory processes, that could influence LF concentration, were not included. In addition, samples were processed in duplicate and quality controls were performed during the ELISA procedure; however, the study did not include an independent analytical validation of the method.

In conclusion, higher LF concentrations were observed in transitional milk and lower concentrations in mature milk during the first postpartum year, with variations among the different lactation periods. These findings provide descriptive information on LF concentration in breast milk in a population from Lima Norte and constitute a reference point for future research.
