# Supplementary material for: Lactoferrin concentration in breast milk during the first postpartum year in Lima Norte, Peru
Source: Rev Peru Med Exp Salud Publica. 2026 Sep 25;43(3):371–8. doi: 10.17843/rpmesp.2026.433.16525 (PMC13718574; doi:10.17843/rpmesp.2026.433.16525)
Supplement: Supplementary file 2 [file rpmesp-43-03-16525-s001.docx]

**Material suplementario**

**Tabla S1.** Cambios en la concentración de lactoferrina desde la etapa de transición a madura, según porcentaje de disminución (n = 44 muestras pareadas de 22 madres).

| Grupo de Disminución | n (%) | Lf Transición (media ± DE) | Lf Madura (media ± DE) | Interpretación |
| --- | --- | --- | --- | --- |
| ≤50% de disminución | 13 (59,1%) | 5,70 ± 1,61  (IC95%: 4,73–6,68) | 3,87 ± 1,06  (IC95%: 3,23–4,51) | Caída leve y sostenida |
| >50% de disminución | 9 (40,9%) | 8,70 ± 2,18  (IC95%: 7,0 – 10,4) | 3,08 ± 0,58  (IC95%: 2,63–3,52) | Alta concentración inicial, caída marcada |

**Tabla S2.** Concentración de lactoferrina según características maternas, neonatales y socioeconómicas.

| **Características** | | **Concentración de lactoferrina (mg/mL)** | | | |  |
| --- | --- | --- | --- | --- | --- | --- |
|  |  |  |  |  |  |  |
|  | **Categorías** | **n = 39** | **Leche transición ± SD** | **n = 53** | **Leche madura ± SD** |  |
|  |  |  |  |  |  |  |
| **Edad de la madre (años)** | ≤19 | 4 | 9,14 ± 2,35 | 6 | 3,86 ± 1,08 |  |
|  | 20–34 | 32 | 6,67 ± 2,04 | 40 | 3,69 ± 1,06 |  |
|  | ≥35 | 3 | 7,88 ± 2,93 | 7 | 3,74 ± 1,25 |  |
| **Edad gestacional (semanas)** |  |  |  |  |  |  |
|  | 37–38 (término temprano) | 15 | 7,27 ± 2,00 | 17 | 3,91 ± 0,96 |  |
|  | 39–40 (término completo) | 23 | 6,89 ± 2,42 | 35 | 3,58 ± 1,09 |  |
|  | 41 (término tardío) | 1 | 6,14 ± 0,00 | 1 | 5,22 ± 0,00 |  |
| **Número de gestaciones** |  |  |  |  |  |  |
|  | 1 gestación | 4 | 8,42 ± 2,81 | 11 | 3,60 ± 0,89 |  |
|  | 2 gestaciones | 21 | 6,54 ± 1,77 | 20 | 3,76 ± 1,10 |  |
|  | ≥3 gestaciones | 14 | 7,33 ± 2,58 | 22 | 3,74 ± 1,15 |  |
| **Tipo de parto** |  |  |  |  |  |  |
|  | Vaginal | 37 | 7,07 ± 2,25 | 49 | 3,68 ± 1,02 |  |
|  | Cesárea | 2 | 6,02 ± 1,58 | 4 | 4,17 ± 1,60 |  |
| **IMC pre-gestacional (Kg/m²)** |  |  |  |  |  |  |
|  | <18,5 (bajo peso) | 0 | 0,00 ± 0,00 | 1 | 4,05 ± 0,00 |  |
|  | 18,5–24,9 (normal) | 19 | 6,93 ± 1,95 | 25 | 4,06 ± 1,02 |  |
|  | 25–29,9 (sobrepeso) | 12 | 7,10 ± 2,43 | 12 | 3,78 ± 1,22 |  |
|  | ≥30 (obesidad) | 8 | 7,10 ± 2,75 | 15 | 3,07 ± 0,78 |  |
| **Educación de la madre** |  |  |  |  |  |  |
|  | Educación primaria | 2 | 6,48 ± 3,04 | 0 | 0,00 ± 0,00 |  |
|  | Educación secundaria | 23 | 7,46 ± 2,41 | 32 | 3,71 ± 1,05 |  |
|  | Educación superior | 6 | 6,86 ± 2,36 | 11 | 3,74 ± 1,15 |  |
|  | No especificado | 8 | 5,97 ± 1,07 | 10 | 3,69 ± 1,13 |  |
| **Género del niño** |  |  |  |  |  |  |
|  | Femenino | 20 | 7,37 ± 2,30 | 27 | 3,58 ± 0,85 |  |
|  | Masculino | 19 | 6,64 ± 2,12 | 26 | 3,86 ± 1,25 |  |
| **Peso al nacer (kg)** |  |  |  |  |  |  |
|  | 2,5–<4,0 (normal) | 37 | 7,07 ± 2,26 | 52 | 3,75 ± 1,05 |  |
|  | ≥4,0 (macrosomía) | 2 | 5,94 ± 0,41 | 1 | 2,17 ± 0,00 |  |
| **Talla al nacer (cm)** |  |  |  |  |  |  |
|  | <48 | 4 | 7,47 ± 2,64 | 9 | 4,05 ± 0,93 |  |
|  | 48–50 | 28 | 6,88 ± 2,22 | 35 | 3,64 ± 1,08 |  |
|  | >50 | 7 | 7,28 ± 2,28 | 9 | 3,68 ± 1,19 |  |
| **Agua y desagüe** |  |  |  |  |  |  |
|  | Sí | 36 | 7,17 ± 2,20 | 48 | 3,72 ± 1,06 |  |
|  | No | 3 | 5,22 ± 1,87 | 5 | 3,64 ± 1,23 |  |
| **Habitantes por vivienda** |  |  |  |  |  |  |
|  | 1–2 | 26 | 6,87 ± 2,05 | 41 | 3,62 ± 0,93 |  |
|  | 3–4 | 7 | 8,37 ± 1,96 | 8 | 3,76 ± 1,46 |  |
|  | ≥5 | 6 | 6,07 ± 2,82 | 4 | 4,57 ± 1,38 |  |
| **Canasta básica de alimentos** |  |  |  |  |  |  |
|  | Sí cubre | 29 | 6,91 ± 2,12 | 46 | 3,62 ± 1,01 |  |
|  | No cubre | 10 | 7,33 ± 2,58 | 7 | 4,31 ± 1,30 |  |
