# Supplementary material for: Lactoferrin concentration in breast milk during the first postpartum year in Lima Norte, Peru
Source: Rev Peru Med Exp Salud Publica. 2026 Sep 25;43(3):371–8. doi: 10.17843/rpmesp.2026.433.16525 (PMC13718574; doi:10.17843/rpmesp.2026.433.16525)
Supplement: Translation

# Translation: Concentración de lactoferrina en leche materna durante el primer año posparto en Lima Norte, Perú

**Authors:** Edison A. Rivera-Fernandez, David Durand, Rafaella Navarro, Yamile Rodriguez-Angeles, Pia S. Pannaraj, Theresa J. Ochoa

**Affiliations:** 1 Instituto de Medicina Tropical “Alexander von Humboldt”, Universidad Peruana Cayetano Heredia, Lima, Perú.; 2 Laboratorios de Investigación y Desarrollo, Universidad Peruana Cayetano Heredia, Lima, Perú.; 3 Division of Infectious Diseases, Department of Pediatrics, University of California, San Diego; La Jolla, CA, United States.; 4 Division of Infectious Diseases, Rady Children’s Hospital, La Jolla, CA, United States.

**Keywords:** Lactoferrina, ELISA, Leche Humana, Lactancia Materna, Periodo de Posparto

## Abstract

La lactoferrina (LF) es una glicoproteína con propiedades antimicrobianas e inmunomoduladoras, presente en la leche materna y fundamental en la protección del lactante. El objetivo fue describir la concentración de LF durante el primer año posparto en Lima Norte, Perú. Se realizó un estudio observacional retrospectivo basado en un análisis secundario de 158 muestras de leche de 83 madres: 39 de leche de transición (7-14 días) y 119 de leche madura (2-12 meses). La LF se cuantificó mediante ELISA. La mediana de LF en leche de transición fue de 6.52 (RIC: 5.16-8.15) mg/mL y en leche madura 1.01 (RIC: 0.80-1.57) mg/mL. En los periodos de leche madura, los valores fueron 3.33 mg/mL (2 meses), 3.27 (3-5 meses), 2.56 (6-7 meses) y 2.08 (8-12 meses). En las 22 madres con muestras pareadas, la concentración media fue de 7.71 ± 2.27 mg/mL en leche de transición y de 3.76 ± 1.06 mg/mL a los 2 meses. La ausencia de muestras suficientes de calostro y la disponibilidad desigual de muestras limitan la caracterización completa de la trayectoria durante el primer año. Los resultados describen un patrón decreciente de la concentración de LF durante el primer año de lactancia.

## INTRODUCCIÓN

La leche materna es reconocida como el alimento ideal para el crecimiento y desarrollo del infante, debido a su contenido de componentes bioactivos como α-lactoalbúmina, β-caseína, inmunoglobulina A secretora (sIgA), lactoferrina (LF) y lisozima ^1^. La concentración de estos biocomponentes proteicos tiende a disminuir a lo largo de las diferentes etapas de la lactancia: el calostro, producido entre el primer y quinto día, presenta las concentraciones más altas; estas disminuyen en la leche de transición (7-14 días) y alcanzan sus niveles más bajos en la leche madura, producida después de los quince días posparto ^2^. Entre estas proteínas, la LF es una de las más abundantes y también presenta variaciones en su concentración según la etapa de lactancia ^3^^-^^6^. Su alta afinidad por el hierro limita su disponibilidad para microorganismos, inhibiendo su crecimiento y confiriéndole un efecto bacteriostático. Este mecanismo, basado en el secuestro de Fe²⁺ y su interacción con estructuras cargadas negativamente, ha sido ampliamente demostrado en diferentes modelos experimentales.

Diversos estudios han evidenciado su actividad frente a bacterias como *Escherichia coli* y *Helicobacter pylori*, así como en modelos celulares infectados con *Legionella pneumophila* y *Mycobacterium tuberculosis*^7^^).^ Además, su actividad ha sido documentada tanto *in vitro* como *in vivo*, mostrando efectos frente a bacterias grampositivas y gramnegativas, e incluso frente a cepas potencialmente resistentes a antibióticos, como *Listeria monocytogenes*, *Klebsiella pneumoniae*, *Salmonella enterica* y *Staphylococcus aureus*^8^^-^^10^. Más allá de su acción antimicrobiana, la LF desempeña un papel clave en la protección del neonato contra infecciones y en el desarrollo del sistema inmunológico. Asimismo, contribuye al equilibrio de la microbiota intestinal, consolidándose como un componente esencial de la leche materna ^11^. La composición fisicoquímica de la leche materna varía durante su maduración y puede verse influenciada por factores maternos y socioeconómicos, particularmente la alimentación y el estado de salud de la madre ^12^.

A pesar de que estudios previos han descrito variaciones en la concentración de LF según la etapa de lactancia, la evidencia disponible corresponde a diferentes poblaciones y contextos geográficos, por lo que sus resultados no necesariamente son extrapolables a la población peruana. En el Perú, la evidencia sobre la concentración de LF en leche materna durante el primer año posparto es limitada, particularmente en muestras de leche de transición y leche madura. Por ello, resulta pertinente caracterizar descriptivamente la concentración de LF durante este periodo en madres atendidas en Lima Norte.

En este contexto, el presente estudio tuvo como objetivo describir la concentración de LF en muestras de leche materna de madres a término (≥37 semanas de gestación) durante el primer año posparto, diferenciando entre leche de transición y leche madura. Las características maternas, neonatales y socioeconómicas se describieron como variables de caracterización de la población.

**MENSAJES CLAVE**

Motivación para realizar el estudio. La lactoferrina es un componente esencial de la leche materna con importantes funciones inmunológicas. Si bien, existen estudios previos sobre su variación según la etapa de lactancia, la evidencia en el contexto peruano es limitada.

Principales hallazgos. Se observó una mayor concentración de lactoferrina en las muestras de leche de transición en comparación con las muestras de leche madura.

Implicancias en salud pública. Estos hallazgos aportan información descriptiva sobre la concentración de lactoferrina en leche de transición y leche madura en población atendida en Lima Norte. La ausencia de muestras de calostro limita la caracterización completa de la variación de la lactoferrina desde las primeras etapas de la lactancia.

## EL ESTUDIO

Se realizó un estudio observacional, descriptivo y retrospectivo en muestras de leche materna de madres de Lima Norte, Perú. El estudio corresponde a un análisis secundario de muestras de leche materna provenientes de un estudio multicéntrico previo ^13^.

El estudio se llevó a cabo entre marzo de 2024 y febrero de 2025 en el laboratorio de la Unidad de Investigación en Infectología Pediátrica (InfectoPed) del Instituto de Medicina Tropical Alexander von Humboldt y en los Laboratorios de Investigación y Desarrollo de la Universidad Peruana Cayetano Heredia, ubicados en el distrito de San Martín de Porres, Lima, Perú.

Se incluyeron 83 madres, con un total de 158 muestras: 39 de leche de transición (7-14 días posparto) y 119 de leche madura (2-12 meses posparto). La disponibilidad de muestras de calostro fue limitada debido a su utilización en el estudio multicéntrico previo, por lo que el análisis se restringió a leche de transición y leche madura.

Se incluyeron participantes del estudio original que autorizaron el uso de sus muestras biológicas y datos para análisis secundarios. Se excluyeron aquellas que no brindaron dicha autorización.

La variable principal fue la concentración de lactoferrina (LF) en leche humana, expresada en mg/mL y determinada mediante la técnica de ELISA. Como variables de caracterización se consideraron el estadio de la leche materna (leche de transición y leche madura), factores maternos (edad materna, edad gestacional, número de gestaciones, tipo de parto e índice de masa corporal), factores neonatales (peso al nacer, talla al nacer y sexo) y factores socioeconómicos (número de habitantes por vivienda, disponibilidad de servicios básicos, área de residencia, nacionalidad y acceso a la canasta básica de alimentos).

Para el análisis, la edad materna se categorizó en ≤19, 20-34 y ≥35 años; la edad gestacional en 37-38 semanas (término temprano), 39-40 semanas (término completo) y 41 semanas (término tardío) ^14^; el número de gestaciones en 1, 2 y ≥3; el tipo de parto en vaginal y cesárea; y el índice de masa corporal pregestacional en <18,5, 18,5-24,9, 25-29,9 y ≥30 kg/m² ^15^. El peso al nacer se categorizó en 2,5-<4,0 kg y ≥4,0 kg ^16^, mientras que la talla al nacer se clasificó en <48, 48-50 y >50 cm. El nivel educativo materno se categorizó en primaria, secundaria, superior y no especificado; el sexo en femenino y masculino; la nacionalidad en Perú y Venezuela; la disponibilidad de servicios básicos en sí y no; y el número de habitantes por vivienda en 1-2, 3-4 y ≥5 personas. Para el componente económico, se calculó el ingreso mensual per cápita del hogar mediante la división del ingreso mensual entre el número de habitantes de la vivienda y se comparó con el valor referencial de la canasta básica de alimentos establecido por el Instituto Nacional de Estadística e Informática (INEI), clasificándose como «sí cubre» cuando el ingreso per cápita fue ≥260 soles y como «no cubre» cuando fue <260 soles ^17^.

Las alícuotas de leche materna fueron descongeladas y sometidas a dos centrifugaciones diferenciales para la obtención del suero de leche. La primera centrifugación se realizó a 1200 × g durante 15 minutos a 4 °C, tras lo cual se separó la fracción acuosa. Esta fracción fue sometida a una segunda centrifugación a 3000 × g durante 15 minutos a 4 °C para obtener el suero de leche, el cual fue almacenado a −70 °C hasta su análisis.

La concentración de LF se determinó mediante ensayo inmunoenzimático (ELISA, Assaypro, EE. UU.), siguiendo las instrucciones del fabricante. Las muestras de suero de leche materna se diluyeron hasta alcanzar una dilución final de de 1:800 000 y se analizaron por duplicado, junto con estándares preparados mediante diluciones seriadas (20-0,313 ng/mL). La absorbancia se midió a 450 nm utilizando un lector de microplacas (Synergy H1m, BioTek Instruments, EE. UU.), y las concentraciones se calcularon mediante interpolación en una curva estándar ajustada con un modelo logístico de cuatro parámetros (4PL), empleando el software MyAssays (versión R10.2), expresándose en mg/mL.

El análisis estadístico se realizó utilizando R (v4.3.2). Las variables continuas se describieron mediante mediana y rango intercuartílico (RIC), y adicionalmente se reportó la media con su intervalo de confianza del 95% (IC 95%) para la concentración de LF. Las variables categóricas se resumieron mediante frecuencias absolutas y relativas. La concentración de LF se describió según la etapa de lactancia y el periodo posparto, considerando las muestras disponibles.

El estudio fue aprobado por el Comité Institucional de Ética de la Universidad Peruana Cayetano Heredia (SIDISI: 213718). Las muestras utilizadas en el presente análisis secundario provinieron de un estudio multicéntrico (SIDISI: 205022), en el cual las participantes otorgaron consentimiento informado para la recolección, almacenamiento y uso futuro de sus muestras biológicas. Para el presente análisis, la información fue manejada de forma confidencial y los datos fueron utilizados sin identificadores personales.

## HALLAZGOS

Se incluyeron 83 madres provenientes de Lima Norte, quienes aportaron un total de 158 muestras de leche materna durante el primer año posparto. Del total de participantes, 31 (37,3%) proporcionaron una sola muestra y 52 (62,6%) dos o más, permitiendo un análisis longitudinal. En relación con la etapa de lactancia, se analizaron 39 muestras de leche de transición (7-14 días) y 119 de leche madura (2-12 meses). No se dispuso de muestras de calostro debido a limitaciones en el volumen recolectado. Entre las muestras de leche madura, 53 correspondieron a los 2 meses ±15 días, 9 a 3-5 meses ±15 días, 39 a 6-7 meses ±15 días y 18 a 8-12 meses ±15 días. En total, 32 madres aportaron muestras en ambas etapas de lactancia.

La edad materna media fue de 27,7 ± 5,9 años, predominando el grupo de 20-34 años (75,9%). La edad gestacional media fue de 38,8 ± 1,0 semanas, con predominio de 39-40 semanas (61,4%), y el parto vaginal fue el más frecuente (92,8%). El índice de masa corporal (IMC) pregestacional medio fue de 26,1 ± 5,0 kg/m², con predominio de la categoría normal (44,6%), seguida de sobrepeso (33,7%). El 51,8% de los recién nacidos fueron de sexo masculino, con un peso medio al nacer de 3,3 ± 0,4 kg y una talla media de 49,1 ± 1,4 cm. En cuanto a las características socioeconómicas, predominó la educación secundaria (50,6%), la nacionalidad peruana (89,2%), la disponibilidad de agua y desagüe (90,4%) y los hogares con 1-2 habitantes (21,7% entre los hogares con número de habitantes especificado). Según el ingreso mensual per cápita respecto al valor referencial de la canasta básica de alimentos, el 49,4% de los hogares se clasificó como «sí cubre» y el 50,6% como «no cubre» (tabla 1).

**Tabla 1:** Características maternas, neonatales y socioeconómicas.

Característica		n=83	%
Edad de la madre (años)			
	≤19	9	10,8
	20-34	63	75,9
	≥35	11	13,3
Edad gestacional (semanas)			
	37-38 (término temprano)	31	37,4
	39-40 (término completo)	51	61,4
	41 (término tardío)	1	1,2
Número de gestaciones			
	1 gestación	14	16,9
	2 gestaciones	36	43,4
	≥3 gestaciones	33	39,7
Tipo de parto			
	Vaginal	77	92,8
	Cesárea	6	7,2
IMC pregestacional (Kg/m²)			
	<18,5 (bajo peso)	2	2,4
	18,5-24,9 (normal)	37	44,6
	25-29,9 (sobrepeso)	28	33,7
	≥30 (obesidad)	16	19,3
Educación de la madre			
	Primaria	14	16,8
	Secundaria	42	50,6
	Superior	15	18,1
	No especificado	12	14,5
Sexo del niño			
	Femenino	40	48,2
	Masculino	43	51,8
Peso al nacer (kg)			
	2,5-<4 (normal)	81	97,6
	≥4,0 (macrosomía)	2	2,4
Talla al nacer (cm)			
	< 48	13	15,7
	48-50	56	67,5
	> 50	14	16,8
Nacionalidad			
	Perú	74	89,2
	Venezuela	9	10,8
Agua y desagüe			
	Sí	75	90,4
	No	8	9,6
Habitantes por vivienda			
	1-2	18	21,7
	3-4	16	19,3
	≥5	11	13,2
	No especificado	38	45,8
Canasta básica de alimentos			
	Sí cubre	41	49,4
	No cubre	42	50,6

La concentración de LF mostró un descenso progresivo a lo largo del periodo de lactancia (figura 1A). En la leche de transición, la mediana fue de 6,52 mg/mL (RIC: 5,47-8,22), mientras que en la leche madura fue de 1,01 mg/mL (RIC: 0,80-1,57). En los diferentes periodos de leche madura, las medianas fueron de 3,33 mg/mL (RIC: 3,05-4,10) a los 2 meses ±15 días, 3,27 mg/mL (RIC: 2,93-4,58) a los 3-5 meses, 2,56 mg/mL (RIC: 2,09-3,23) a los 6-7 meses y 2,26 mg/mL (RIC: 1,36-3,28) a los 8-12 meses (tabla 2; figura 1B).

**Figura 1:** Distribución de la concentración de lactoferrina según: A) días posparto y B) etapas de lactancia, en muestras de leche materna (n=158).

**Tabla 2:** Concentración de lactoferrina (mg/ml) en leche de transición y madura en diferentes periodos posparto (n=158).

Tipo de leche	n	Concentración de lactoferrina (mg/mL)			
		Mediana (RIC)	Media (IC 95%)	Min	Max
Leche de transición (7-14 días)	39	6,52 (5,47-8,22)	7,02 (6,30-7,73)	3,15	11,49
Leche madura (2 meses ±15 días)	53	3,33 (3,05-4,10)	3,72 (3,42-4,01)	2,17	5,94
Leche madura (3-5 meses ±15 días)	9	3,27 (2,93-4,58)	3,64 (2,70-4,57)	1,43	5,11
Leche madura (6-7 meses ±15 días)	39	2,56 (2,09-3,23)	2,73 (2,42-3,04)	0,88	4,98
Leche madura (8-12 meses ±15 días)	18	2,26 (1,36-3,28)	2,24 (1,66-2,81)	0,43	4,08

RIC: rango intercuartil, IC 95%: intervalo de confianza al 95%.

En 32 madres con múltiples muestras (n=126), se observó un descenso progresivo de la concentración de LF desde la leche de transición hacia los diferentes periodos de leche madura (figura 2). De estas 32 madres, 22 contaron con una muestra de leche de transición y una muestra de leche madura correspondiente al periodo de 2 meses ±15 días (figura 3A), con un total de 44 muestras para el análisis pareado (figura 3B). En este subgrupo, la concentración media de LF fue de 7,71 ± 2,27 mg/mL en la leche de transición y de 3,76 ± 1,06 mg/mL en la leche madura. Las medianas fueron de 7,28 y 3,42 mg/mL, respectivamente. El 59,1% de las madres presentó una disminución ≤50%, mientras que el 40,9% presentó una disminución >50% (material suplementario tabla S1).

**Figura 2:** Concentración de lactoferrina (mg/mL) en leche de transición y madura en madres con seguimiento longitudinal (n=126 muestras pareadas de 32 madres).

**Figura 3:** Comparación de la concentración de lactoferrina (mg/mL) entre leche de transición y leche madura (2 meses ±15 días). A) Comparación entre grupos independientes de leche de transición (n=39) y leche madura (n=53). B) Comparación pareada en 22 madres con una muestra de leche de transición y una muestra de leche madura (22 pares; n=44 muestras).

El análisis descriptivo de las concentraciones de LF según las características maternas, neonatales y socioeconómicas se presenta en el material suplementario tabla S2.

## DISCUSIÓN

En este estudio se evidenció una disminución progresiva de la concentración de LF durante el primer año posparto, desde la leche de transición hasta la leche madura. Este patrón ha sido ampliamente descrito en la literatura como parte de un proceso adaptativo de la leche materna, orientado a responder a los requerimientos inmunológicos cambiantes del lactante ^2^^,^^3^.

Este fenómeno también ha sido documentado en poblaciones de neonatos prematuros, donde las concentraciones iniciales de LF tienden a ser más elevadas, especialmente en madres de recién nacidos y prematuros, como mecanismo compensatorio frente a la inmadurez inmunológica neonatal ^18^^,^^19^. La magnitud de las concentraciones puede variar según las características de la población.

Al comparar estos resultados con otros estudios, es necesario considerar las diferencias entre las poblaciones estudiadas. Turin *et al*. reportaron concentraciones considerablemente más altas de LF tanto en leche de transición como en leche madura; sin embargo, su población estuvo conformada principalmente por madres de recién nacidos prematuros y de bajo peso, condiciones asociadas con concentraciones más elevadas de LF ^20^^,^^21^. En contraste, el presente estudio incluyó madres de recién nacidos a término. Esta diferencia limita la comparabilidad directa entre ambas poblaciones.

En poblaciones de recién nacidos a término, los hallazgos son más consistentes con nuestros resultados. Mastromarino *et al*. y Hernández Pérez documentaron concentraciones iniciales entre 5,4 y 7,0 mg/mL, con valores en leche madura entre 1,7 y 2,3 mg/mL ^4^^,^^22^, rangos similares a los observados en nuestra población en etapas posteriores. Asimismo, Zhang *et al*. reportaron una disminución progresiva durante los primeros meses de lactancia, aunque con valores absolutos menores, posiblemente debido a diferencias en unidades de medida, técnicas analíticas o características poblacionales. Aunque la magnitud difiere, la tendencia descendente es comparable ^23^.

En nuestro caso, la reducción desde 6,52 mg/mL en leche de transición hasta 2,26 mg/mL entre los 8 y 12 meses evidencia el carácter dinámico de la concentración de LF durante la lactancia ^3^. Además, estudios longitudinales han descrito que, tras esta disminución inicial, la LF puede estabilizarse o incluso incrementarse ligeramente en etapas posteriores de la lactancia ^24^.

Diversos estudios han descrito variaciones en la concentración de LF en relación con características maternas, neonatales y contextuales ^(^^25^^-^^27^. Sin embargo, las diferencias en las poblaciones estudiadas, el momento de obtención de las muestras y las metodologías empleadas dificultan establecer comparaciones directas con los resultados descriptivos del presente estudio.

En este contexto, otros estudios aportan evidencia complementaria. Czosnykowska-Łukacka *et al*. observaron incrementos en la LF en lactancias prolongadas ^28^, mientras que Kulesza-Brończyk *et al*. reportaron variaciones de la concentración de LF según características neonatales en etapas tempranas ^29^. Por su parte, Rai *et al*., en una revisión sistemática, describieron mayores concentraciones de LF en etapas iniciales y una disminución posterior hasta valores correspondientes a la leche madura ^30^.

Entre las limitaciones del estudio, se incluyen el tamaño muestral reducido y la relativa homogeneidad de la población. Asimismo, no todas las madres aportaron muestras en todas las etapas, generando una distribución desigual de las observaciones entre los periodos posparto y limitando su representatividad. Además, no se incluyeron variables potencialmente relevantes, como la dieta materna y los procesos inflamatorios, que podrían influir en la concentración de LF. Además, las muestras fueron procesadas por duplicado y se realizaron controles de calidad durante el procedimiento ELISA; sin embargo, el estudio no incluyó una validación analítica independiente del método.

En conclusión, se observaron mayores concentraciones de LF en la leche de transición y menores concentraciones en la leche madura durante el primer año posparto, con variaciones entre los diferentes periodos de la lactancia. Estos hallazgos aportan información descriptiva sobre la concentración de LF en leche materna en una población de Lima Norte y constituyen un punto de referencia para futuras investigaciones.
